# Gameful experience, exercise identity, and exercise adherence among Chinese university students: A moderated chain mediation model

**DOI:** 10.1371/journal.pone.0353100

**Published:** 2026-10-06

**Authors:** Jingjing Song, Jiaying Li, Dan Li

**Affiliations:** 1 College of Physical Education and Health Management, Chongqing University of Education, Chongqing, China; 2 School of Physical Education, Chongqing University of Posts and Telecommunications, Chongqing, China; 3 School of Physical Education, Wuhan University of Science and Technology, Wuhan, Hubei, China; Farhangian Teacher Education University: Farhangian University, IRAN, ISLAMIC REPUBLIC OF

## Abstract

**Background:**

Gamified fitness applications have grown in popularity, yet it remains unclear how gameful experience, defined as users’ subjective perception of game-like engagement during app-supported exercise, is associated with exercise adherence.

**Methods:**

A cross-sectional survey was conducted among *N* = 300 Chinese university students who were active users of gamified fitness applications. Data were collected via an online questionnaire platform (Questionnaire Star) from March 5 to April 5, 2026. Participants completed validated measures of gameful experience (GAMEX), exercise identity (EI), exercise habit formation (EHF), intolerance of uncertainty (IU), and exercise adherence (EA). A moderated chain mediation model was tested using hierarchical regression analysis and bias-corrected bootstrap resampling (5,000 iterations). An indirect effect was considered statistically different from zero when its 95% confidence interval excluded zero.

**Results:**

Gameful experience was positively associated with exercise adherence (β = .264, *p* < .001). The observed associations were consistent with three indirect pathways: GAMEX → EI → EA (effect = .065, 95% CI [.020, .114]); GAMEX → EHF → EA (effect = .043, 95% CI [.002, .087]); and GAMEX → EI → EHF → EA (effect = .085, 95% CI [.054, .123]). IU moderated the GAMEX–EI association (β = .237, *p* < .001, $\Delta R^{2}$ = .036); the estimated chain indirect effect ranged from .034 (95% CI [.001, .072]) at low IU to .136 (95% CI [.080, .201]) at high IU. These estimates describe conditional associations and do not establish causal or temporal ordering.

**Conclusions:**

The findings are consistent with the hypothesized ordering of gameful experience, exercise identity, exercise habit formation, and adherence, while alternative causal orderings remain possible. The moderation result suggests that IU may be a useful boundary condition to examine in longitudinal and experimental studies of identity-supportive app features.

## Introduction

Insufficient physical activity among university students remains a public health concern, and maintaining regular exercise after initial uptake is particularly difficult [1]. Gamified fitness applications embed points, badges, leaderboards, challenges, and progress feedback in exercise platforms [2]. Randomized trials and quantitative reviews suggest that these interventions can increase physical activity or app engagement, but their effects vary by feature, population, and follow-up period [3–6]. In particular, evidence for maintenance after gamification ends is mixed. Much of the literature focuses on app use, behavioral intentions, or short-term activity rather than sustained exercise [3,4,6]. Exposure to game elements alone may therefore be insufficient to explain continued participation. Users differ in the psychological engagement that they experience while exercising with an app. This subjective response, termed *gameful experience* and measured with the GAMEX scale, is distinct from an objective count of app features [2]. However, the psychological variables linking gameful experience with exercise adherence remain incompletely specified.

Exercise identity and exercise habit formation may help account for this association. Exercise identity refers to the extent to which the “exerciser” role forms part of a person’s self-concept [7]. Stronger exercise identity has been associated with later activity and maintenance in longitudinal and intervention studies [8–10], and identity-focused app design has been proposed as a way to connect repeated activity with self-definition [11]. Findings are not uniform. During behavioral weight-loss treatment, changes in exercise identity were not reliably coupled with changes in accelerometer-measured exercise [12]. Exercise habit formation refers here to the automatic initiation of exercise [13]. Automaticity develops through repetition in stable contexts and predicts physical activity beyond reflective intentions [14–17]. Identity theory and habit research suggest a possible sequence: people who identify more strongly as exercisers may organize activity in stable settings, and repeated performance in those settings may support automaticity [17,18]. This identity-to-habit ordering offers one explanation for the association between gameful experience and adherence [19]. Yet most explanatory models examine one mediator at a time [20], and cross-sectional evidence cannot establish whether identity precedes habit.

Intolerance of uncertainty (IU) may qualify the first part of this proposed sequence. Gamified fitness applications combine uncertain elements, including variable challenges and competitive outcomes, with predictable feedback such as progress bars, streak counts, and badges. Prospective IU is associated with attempts to obtain information and reduce uncertainty, and people higher in prospective IU may seek more feedback when uncertain outcomes are personally relevant [21–23]. Clear progress feedback may provide “structured certainty” and strengthen the association between gameful experience and exercise identity among users higher in IU [24–26]. A competing response is also plausible because uncertain rewards or social comparison may increase distress or avoidance. The present survey could not distinguish these explanations because it did not manipulate app features. We examined Chinese university students, whose physical fitness and regular exercise rates remain a public health concern [1]. Drawing on identity theory, habit formation theory, and the IU literature, we tested whether the observed covariance pattern was consistent with a moderated chain mediation model linking gameful experience (GAMEX) with exercise adherence (EA) through exercise identity (EI) and exercise habit formation (EHF), with IU moderating the GAMEX–EI association. The directional hypotheses specify the theoretical model but do not establish temporal order:

**Hypothesis 1 (H1):** Gameful experience is positively associated with exercise adherence.
**Hypothesis 2 (H2):** Exercise identity mediates the relationship between gameful experience and exercise adherence (GAMEX → EI → EA).
**Hypothesis 3 (H3):** Exercise habit formation mediates the relationship between gameful experience and exercise adherence (GAMEX → EHF → EA).
**Hypothesis 4 (H4):** Exercise identity and habit formation constitute a sequential chain mediation pathway (GAMEX → EI → EHF → EA).
**Hypothesis 5 (H5):** The positive association between gameful experience and exercise identity varies as a function of intolerance of uncertainty and is stronger at higher levels of IU.

## Participants and methods

### Participants and data collection

This cross-sectional survey examined associations among the study variables; it cannot establish causal effects or temporal ordering. Data were collected from March 5 to April 5, 2026 through Questionnaire Star (Wenjuanxing), a Chinese online survey platform, and distributed to students at multiple Chinese universities. Eligibility required participants to be current university students, current users of an exercise app with game-like features, and willing to provide informed consent. Participation was voluntary. Respondents could stop by closing the survey before submission; incomplete questionnaires were not included in the analytic file. The survey did not collect chronological age, which prevents reporting an age mean or range and is acknowledged as a limitation.

A total of 320 completed questionnaires were received. Data cleaning excluded 20 submissions with a repetitive response pattern exceeding 70% or an implausibly short or long completion time; 300 responses remained (93.75%). The sample comprised 148 men (49.3%) and 152 women (50.7%). By academic year, 76 participants (25.3%) were in Year 1, 87 (29.0%) in Year 2, 92 (30.7%) in Year 3, and 45 (15.0%) in Year 4 or above. App-use duration was less than three months for 36 participants (12.0%), 3–6 months for 77 (25.7%), 6–12 months for 107 (35.7%), and more than one year for 80 (26.7%). The reported apps were Keep (*n* = 94), Codoon/Gu Dong (*n* = 77), Yuedongquan (*n* = 67), Nike (*n* = 37), and other apps (*n* = 25). Weekly use ranged from less than once (*n* = 20, 6.7%) to five or more times (*n* = 94, 31.3%).

This study received ethical approval from the Institutional Review Board of Chongqing University of Education (Approval No.: CQUE-LL20260306002, approved March 4, 2026). All participants provided written informed consent prior to completing the questionnaire and were assured of data confidentiality and the voluntary nature of their participation. All procedures were conducted in accordance with the Declaration of Helsinki.

Fig 1 summarizes recruitment, screening, measurement, and analysis.

**Fig 1:**
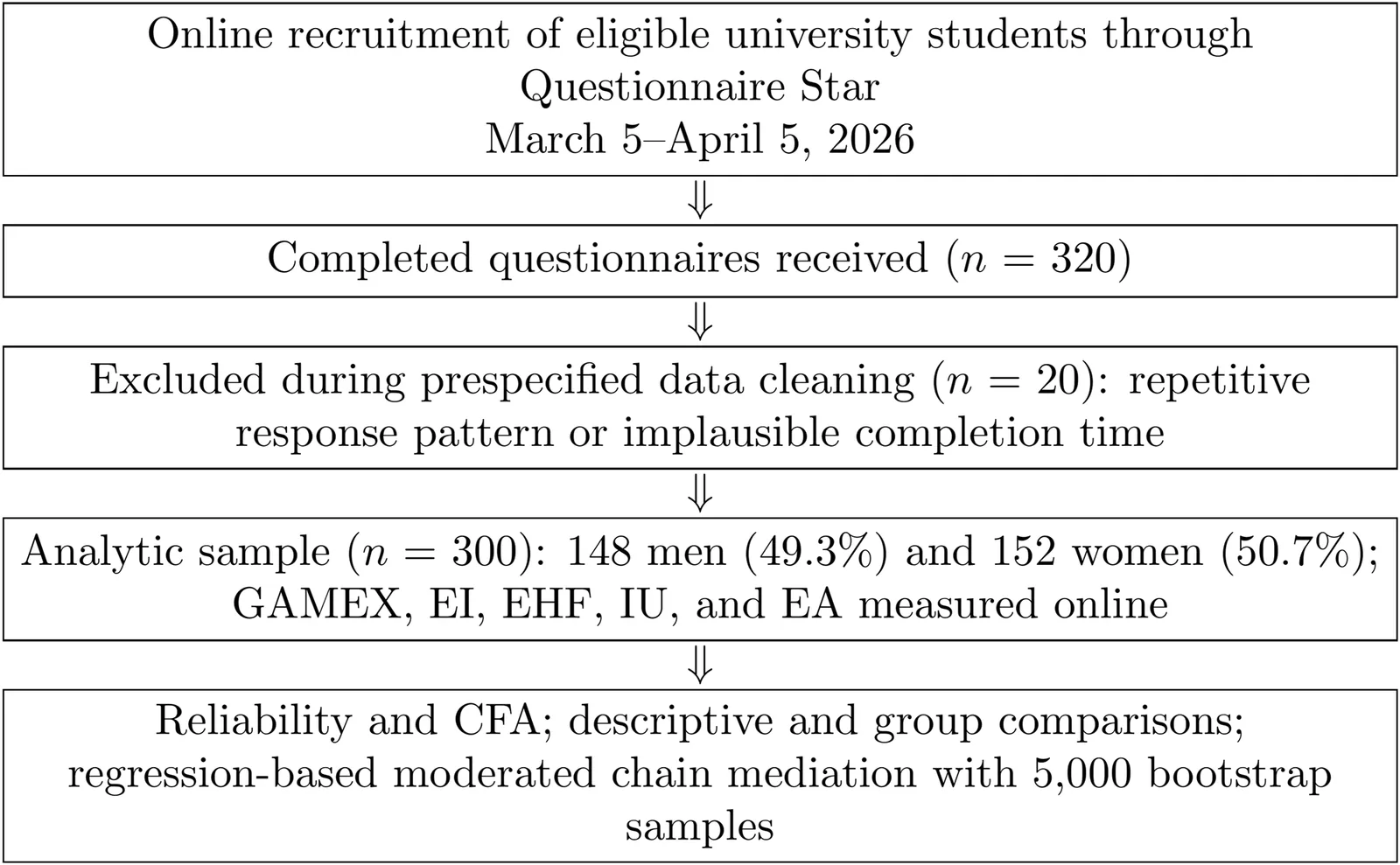
Research activities, participant flow, measurements, and analyses. The analytic sample comprised 148 men (49.3%) and 152 women (50.7%). Abbreviations: GAMEX, Gameful Experience; EI, Exercise Identity; EHF, Exercise Habit Formation; IU, Intolerance of Uncertainty; EA, Exercise Adherence.

### Measures

#### Gameful experience (GAMEX).

Gameful experience was assessed using the Gameful Experience Scale (GAMEX; [2]), a 27-item instrument capturing six dimensions: Enjoyment (6 items), Absorption (6 items), Creative Thinking (4 items), Activation (4 items), Absence of Negative Affect (3 items, reverse-scored), and Dominance (4 items). Participants responded on a 7-point Likert scale (1 = strongly disagree to 7 = strongly agree). A composite mean score was computed, with higher scores reflecting stronger gameful experience. Cronbach’s α in the present sample was .982.

#### Exercise identity (EI).

Exercise identity was measured using the Exercise Identity Scale (EIS; [7]), a 9-item instrument assessing the degree to which individuals incorporate the “exerciser” role into their self-concept (e.g., “I consider myself an exerciser”). Items were rated on a 7-point scale (1 = strongly disagree to 7 = strongly agree), with higher mean scores indicating stronger exercise identity. Cronbach’s α = .947.

#### Exercise habit formation (EHF).

Exercise habit formation was operationalized using the Self-Report Behavioural Automaticity Index (SRBAI; [13]), a 4-item subscale assessing the automaticity of exercise behavior (e.g., “For me, exercising is something I do automatically”). Items were rated on a 7-point scale, with higher mean scores indicating greater behavioral automaticity. Cronbach’s α = .897.

#### Intolerance of uncertainty (IU).

Intolerance of uncertainty was measured using the Chinese version of the 12-item Intolerance of Uncertainty Scale (IUS-12; [21,27]), which comprises two subscales: Prospective Anxiety (IUS-P, 7 items) and Inhibitory Anxiety (IUS-I, 5 items). Responses were given on a 5-point scale (1 = not at all characteristic of me to 5 = entirely characteristic of me), with higher mean scores indicating greater intolerance of uncertainty. Cronbach’s α = .954.

#### Exercise adherence (EA).

Exercise adherence was assessed using a 6-item scale adapted from the Exercise Adherence Rating Scale (EARS; [28]), with items contextually modified for Chinese university students (e.g., “I am able to maintain exercise at my self-set frequency”). Items 4 and 6 were reverse-scored. Responses used a 5-point scale (1 = not at all true to 5 = completely true), with higher mean scores indicating greater adherence. Cronbach’s α = .919.

All English-language items were translated into Chinese and back-translated to check semantic correspondence. No separate pilot study was conducted before the main survey. We therefore evaluated internal consistency and factorial validity in the full analytic sample and acknowledge the absence of an independent pretest as a measurement limitation.

### Data analysis

Descriptive statistics, Cronbach’s α, Harman’s single-factor test, independent-samples tests by gender, and one-way analyses of variance by academic year and app-use duration were conducted in SPSS Version 26.0 (IBM Corp., Armonk, NY). We also compared participants reporting less than three months of app use with those reporting more than one year. CFA was performed in R (Version 4.3.2) with *lavaan* (Version 0.6–17) [29]. Item parcels were used to represent the prespecified dimensions of multidimensional scales and to keep the measurement model parsimonious relative to *N* = 300. Following published recommendations, parceling was applied only after examining internal consistency and the intended dimensional structure [30]. The five-factor model was compared with four-, three-, and one-factor alternatives using $\chi^{2}$/df, CFI, TLI, and RMSEA. PROCESS Version 4.2 for SPSS [31] was selected because the hypotheses concerned conditional indirect effects among observed composite scores and PROCESS directly estimates serial mediation, interactions, simple slopes, and bootstrap confidence intervals. For observed-variable models, PROCESS and path analysis in SEM generally yield equivalent coefficient estimates [32]; CFA was used separately to examine measurement structure. Bias-corrected bootstrap confidence intervals used 5,000 resamples. Continuous variables were standardized before computing interaction terms.

## Results

### Common method bias and descriptive statistics

Harman’s single-factor test was conducted on all 58 items. The first unrotated factor accounted for 41.08% of the total variance. Although this value is below the conventional 50% heuristic, Harman’s test has limited sensitivity and cannot establish the absence of common method variance [33]. Because all variables were self-reported online at one time point, common method bias remains a plausible alternative explanation for part of the observed covariance.

Descriptive statistics and reliability coefficients for all variables are presented in Table 1. The GAMEX composite mean was 4.754 (*SD* = 0.764); among the six subdimensions, Absence of Negative Affect had the highest score (*M* = 5.753) and Activation the lowest (*M* = 4.361). Exercise identity (*M* = 4.607, *SD* = 0.815), habit formation (*M* = 4.088, *SD* = 0.914), and exercise adherence (*M* = 3.551, *SD* = 0.569) all fell at moderate-to-high levels on their respective scales. The IU mean was 2.876 (*SD* = 0.521). Absolute skewness values were all below 1.0 and absolute kurtosis values below 2.0, supporting approximate normality. Cronbach’s α ranged from .863 to .982, indicating acceptable to excellent internal consistency.

**Table 1:** Descriptive statistics, reliability, and common method bias test (*N* = 300).

Variable	Items	M	SD	Skew	Kurt	α	Role
*Gameful Experience (GAMEX, independent variable, 7-point scale)*							
Enjoyment (ENJ)	6	4.942	0.805	−0.10	−0.17	.930	Subdimension of X
Absorption (ABS)	6	4.548	0.806	−0.19	−0.24	.919	
Creative Thinking (CRT)	4	4.640	0.804	−0.05	−0.33	.881	
Activation (ACT)	4	4.361	0.809	−0.03	−0.24	.885	
Absence of Neg. Affect (ANA)	3	5.753	0.710	−0.29	−0.31	.863	
Dominance (DOM)	4	4.541	0.809	−0.11	−0.14	.893	
**GAMEX total (X)**	**27**	**4.754**	**0.764**	**−0.13**	**−0.31**	**.982**	Independent variable (X)
*Mediators and Outcome*							
Exercise Identity (EI, M1, 7-point)	9	4.607	0.815	0.15	−0.27	.947	Mediator 1 (M1)
Exercise Habit Formation (EHF, M2, 7-point)	4	4.088	0.914	0.06	−0.06	.897	Mediator 2 (M2)
Exercise Adherence (EA, Y, 5-point)	6	3.551	0.569	−0.47	−0.13	.919	Outcome (Y)
*Moderator*							
Intolerance of Uncertainty (IU, W, 5-point)	12	2.876	0.521	0.03	1.30	.954	Moderator (W)

Harman’s single-factor test yielded a first-factor variance of 41.08%, below the conventional 50% heuristic; however, this test cannot rule out common method variance. Absolute skewness < 1 and absolute kurtosis < 2 for all variables, supporting approximate normality. α = Cronbach’s α internal consistency coefficient. GAMEX = Gameful Experience; EI = Exercise Identity; EHF = Exercise Habit Formation; IU = Intolerance of Uncertainty; EA = Exercise Adherence.

### Demographic and app-use group comparisons

No statistically significant differences were detected across gender, academic year, or the four app-use-duration categories for GAMEX, EI, EHF, IU, or EA (all *p* > .15). Gender effects were small (|*d*| = .032–.144), and academic-year and duration effects were also small ($\eta^{2}$ = .001–.018). The targeted comparison of respondents with less than three months of use (*n* = 36) versus more than one year (*n* = 80) was likewise non-significant for GAMEX (*p* = .330), EI (*p* = .895), EHF (*p* = .506), IU (*p* = .174), and EA (*p* = .553). These exploratory comparisons do not indicate substantial subgroup heterogeneity in the study variables.

### Correlation analysis

Pearson correlation coefficients among all primary variables are reported in Table 2. GAMEX was positively correlated with EI (*r* = .372, *p* < .001), EHF (*r* = .290, *p* < .001), and EA (*r* = .264, *p* < .001). The EI–EHF correlation was the strongest among the variable pairs (*r* = .546, *p* < .001), indicating a close association but not their temporal ordering. EI (*r* = .426, *p* < .001) and EHF (*r* = .538, *p* < .001) were also correlated with EA. IU had no significant zero-order correlation with any primary variable (all *p* > .05); this does not preclude an interaction effect.

**Table 2:** Descriptive statistics and correlation matrix for primary variables (*N* = 300).

Variable	M	SD	1	2	3	4
1. Gameful Experience (GAMEX)	4.754	0.764	—			
2. Exercise Identity (EI)	4.607	0.815	.372***	—		
3. Exercise Habit Formation (EHF)	4.088	0.914	.290***	.546***	—	
4. Intolerance of Uncertainty (IU)	2.876	0.521	−.012	−.097	−.092	—
5. Exercise Adherence (EA)	3.551	0.569	.264***	.426***	.538***	−.022

*p < .05, **p < .01, ***p < .001 (two-tailed). GAMEX = Gameful Experience (X); EI = Exercise Identity (M1); EHF = Exercise Habit Formation (M2); IU = Intolerance of Uncertainty (W); EA = Exercise Adherence (Y).

### Confirmatory factor analysis

CFA results comparing the hypothesized five-factor model against alternative structures are reported in Table 3. The five-factor model demonstrated excellent fit: $\chi^{2}$/df = 1.147, CFI = .997, TLI = .996, RMSEA = .022, all meeting or surpassing standard thresholds ($\chi^{2}$/df < 3, CFI > .95, TLI > .95, RMSEA < .06; [34]). In contrast, the four-factor model (merging EI and EHF) showed substantially poorer fit ($\chi^{2}$/df = 5.081, RMSEA = .117), with further deterioration in three-factor (RMSEA = .204) and single-factor (RMSEA = .262) models. Standardized factor loadings for the five-factor model ranged from .791 to 1.000 (all *p* < .001), supporting convergent validity [35].

**Table 3:** Confirmatory factor analysis: model fit comparison (*N* = 300).

Model	$\chi^{2}$	df	$\chi^{2}$/df	CFI	TLI	RMSEA	AIC	BIC
M1: Five-factor (hypothesized) ✓	143.40	125	1.147	.997	.996	.022	91.04	261.42
M2: Four-factor (EI + EHF merged)	655.44	129	5.081	.913	.896	.117	79.63	235.19
M3: Three-factor (GAMEX + EI + EHF merged)	1780.00	132	13.485	.727	.683	.204	66.13	210.58
M4: Single-factor	2908.26	135	21.543	.540	.479	.262	52.61	185.95

Acceptable fit criteria: CFI > .95, TLI > .95, RMSEA < .06, $\chi^{2}$/df < 3 [34]. ✓ indicates the best-fitting model. M1 = five-factor hypothesized model (GAMEX, EI, EHF, IU, EA as separate factors), supporting discriminant validity across all five constructs.

Standardized factor loadings for the five-factor model are presented in Table 4.

**Table 4:** Standardized factor loadings for the five-factor model (*N* = 300).

Construct	Indicator	λ (standardized)	*z*
Gameful Experience (GAMEX, X)	Enjoyment (ENJ)	.970	—
	Absorption (ABS)	.961	44.84***
	Creative Thinking (CRT)	.944	39.77***
	Activation (ACT)	.948	40.71***
	Absence of Neg. Affect (ANA)	.916	33.91***
	Dominance (DOM)	.951	41.71***
Exercise Identity (EI, M1)	Parcel 1 (items 1–3)	.922	—
	Parcel 2 (items 4–6)	.939	28.79***
	Parcel 3 (items 7–9)	.920	27.32***
Exercise Habit Formation (EHF, M2)	Item 1	.791	—
	Item 2	.837	15.84***
	Item 3	.829	15.64***
	Item 4	.863	16.42***
Intolerance of Uncertainty (IU, W)	Prospective subscale (IUS-P)	.912	—
	Inhibitory subscale (IUS-I)	1.000	5.49***
Exercise Adherence (EA, Y)	Parcel 1 (items 1–2)	.890	—
	Parcel 2 (items 3–4)	.873	20.62***
	Parcel 3 (items 5–6)	.891	21.29***

λ = standardized factor loading. The first indicator per construct serves as the reference indicator. All loadings > .79, *p* < .001. ***p < .001.

### Moderated chain mediation analysis

#### Main effects and mediation paths (H1–H4).

The conceptual model is illustrated in Fig 2. Regression results are presented in Table 5, and bootstrap indirect-effect estimates in Table 6. After adjustment for IU, GAMEX was associated with EI (β = .394, *SE* = .053, *t* = 7.449, *p* < .001; Model 1). EI was associated with EHF (β = .509, *SE* = .052, *t* = 9.783, *p* < .001; Model 2), and EHF was associa*t*ed with EA (β = .426, *SE* = .058, *t* = 7.399, *p* < .001; Model 3). The coefficient relating GAMEX to EHF was not significant af*t*er EI was included (β = .100, *p* = .055). Likewise, the coefficient relating GAMEX to EA was not significant after bo*t*h mediators were included (β = .080, *p* = .125). Within the specified model, this is an indirect-only pattern; it does not prove a causal mechanism.

**Table 5:** Regression results for chain mediation and moderation (*N* = 300).

	Model 1: EI (M1)				Model 2: EHF (M2)				Model 3: EA (Y)			
Predictor	β	SE	t	p	β	SE	t	p	β	SE	t	p
GAMEX (X)	.394	.053	7.449	<.001	.100	.052	1.929	.055	.080	.052	1.539	.125
IU (W)	−.060	.053	−1.133	.258	—	—	—	—	—	—	—	—
GAMEX × IU	.237	.066	3.609	<.001	—	—	—	—	—	—	—	—
EI (M1)	—	—	—	—	.509	.052	9.783	<.001	.163	.059	2.751	.006
EHF (M2)	—	—	—	—	—	—	—	—	.426	.058	7.399	<.001
*R* ^2^	.183				.307				.320			
Adj. *R*^2^	.172				.300				.311			
*F*	22.109***				65.868***				46.455***			

All variables were standardized (*z*-scored) prior to analysis. β = standardized regression coefficient. Model 1 includes the moderation interaction term; $\Delta R^{2}$ (interaction term) = .036. ***p < .001, **p < .01. GAMEX = Gameful Experience (X); EI = Exercise Identity (M1); EHF = Exercise Habit Formation (M2); IU = Intolerance of Uncertainty (W); EA = Exercise Adherence (Y).

**Table 6:** Decomposition of moderated indirect effects (Bootstrap = 5,000, *N* = 300).

Indirect effect pathway	IU level	Effect	SE	LLCI	ULCI	Sig.
Path 1: GAMEX (X) → EI (M1) → EA (Y)	Low (−1 SD)	.026	.017	<.001	.067	Yes
	Mean	.065	.024	.020	.114	Yes
	High (+1 SD)	.102	.038	.032	.179	Yes
Path 2: GAMEX (X) → EHF (M2) → EA (Y)	Low (−1 SD)	.043	.022	<.001	.087	Yes
	Mean	.043	.022	.002	.087	Yes
	High (+1 SD)	.043	.022	.003	.087	Yes
Path 3 (chain): X → M1 → M2 → Y	Low (−1 SD)	.034	.018	.001	.072	Yes
	Mean	.085	.018	.054	.123	Yes
	High (+1 SD)	.136	.031	.080	.201	Yes

Bias-corrected Bootstrap (5,000 resamples). Effect = point estimate; LLCI/ULCI = lower/upper bounds of the 95% confidence interval. A CI excluding zero indicates significance. Shaded rows = serial chain mediation pathway. GAMEX = Gameful Experience (X); EI = Exercise Identity (M1); EHF = Exercise Habit Formation (M2); IU = Intolerance of Uncertainty (W); EA = Exercise Adherence (Y).

**Fig 2:**
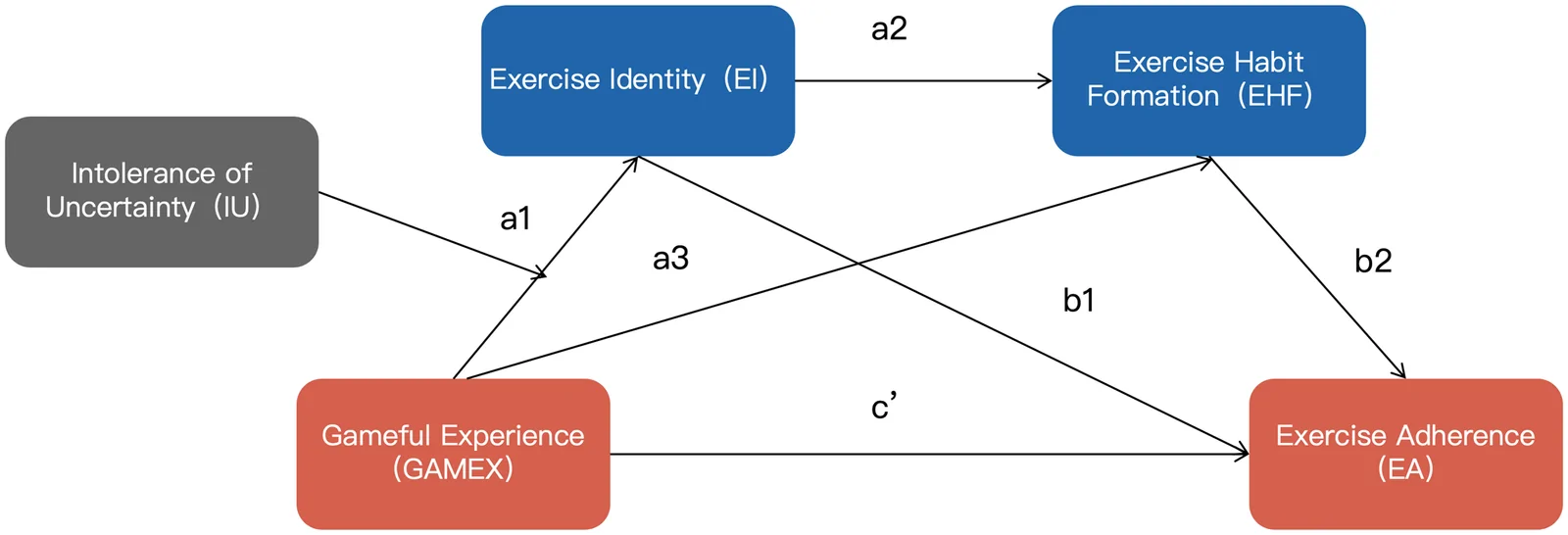
Conceptual model of the moderated chain mediation framework. The arrow from IU to path a1 denotes moderation of the GAMEX–EI association. Abbreviations: GAMEX, Gameful Experience; EI, Exercise Identity; EHF, Exercise Habit Formation; EA, Exercise Adherence; IU, Intolerance of Uncertainty.

Bootstrap estimates (Table 6) were non-zero for all three hypothesized indirect paths at mean IU: GAMEX → EI → EA, effect = .065, 95% CI [.020, .114]; GAMEX → EHF → EA, effect = .043, 95% CI [.002, .087]; and GAMEX → EI → EHF → EA, effect = .085, 95% CI [.054, .123]. These results support H2–H4 at the level of statistical associations.

#### Moderation analysis (H5).

The IU × GAMEX interaction was associated with EI in Model 1 (β = .237, *SE* = .066, *t* = 3.609, *p* < .001), with $\Delta R^{2}$ = .036, supporting H5. The IU main-effect coefficient was not significant (β = −.060, *p* = .258). Simple slopes (Fig 3) showed a stronger posi*t*ive GAMEX–EI association at high IU (β = .632, *p* < .001) than at mean IU (β = .394, *p* < .001) or low IU (β = .157, *p* < .001).

**Fig 3:**
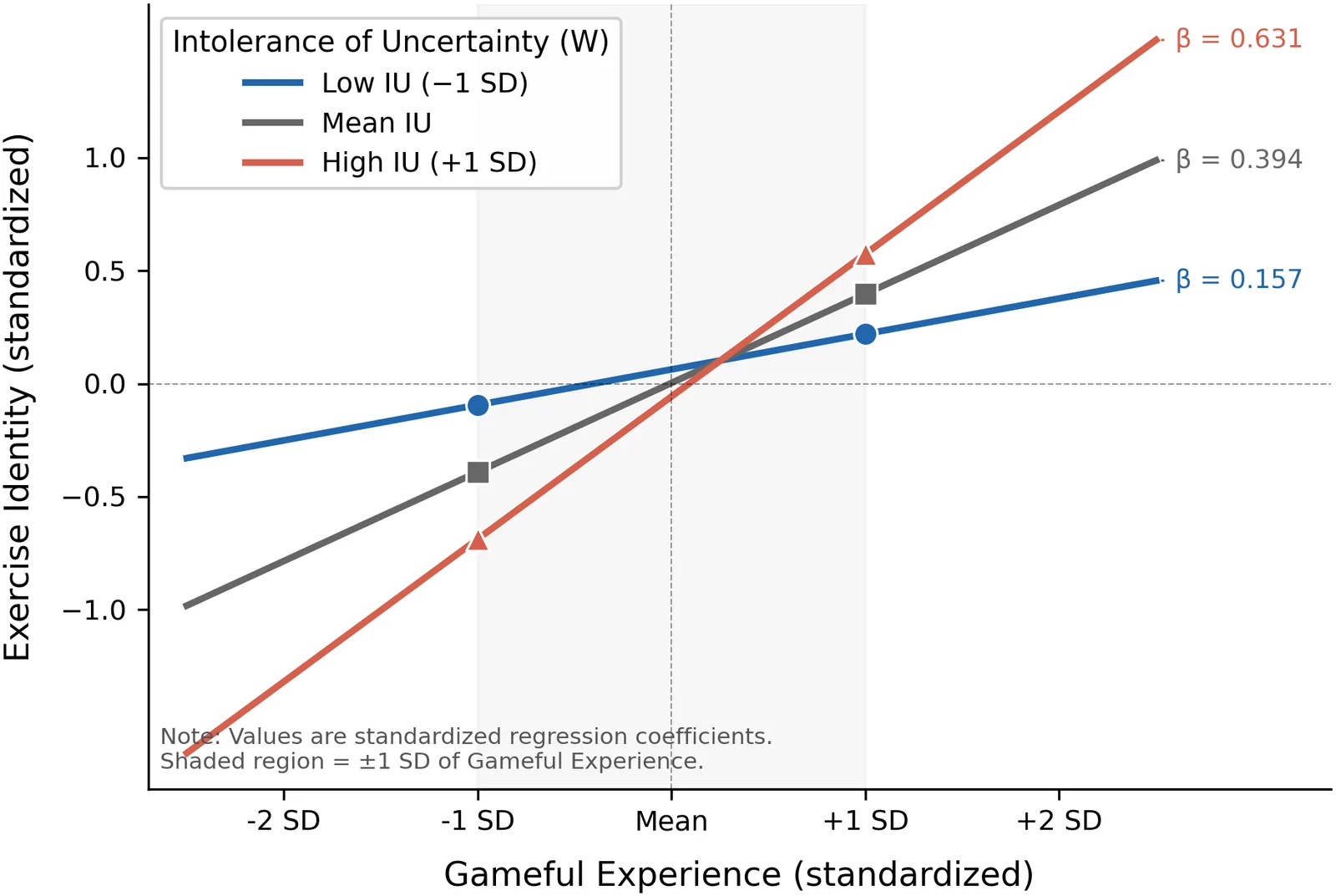
Simple slopes for the moderating effect of intolerance of uncertainty (IU) on the gameful experience–exercise identity association. Three simple slopes are plotted at low IU (−1 SD), mean IU, and high IU (+1 SD). Steeper slopes at higher IU levels indicate that individuals with greater intolerance of uncertainty show a stronger positive association between gameful experience and exercise identity. All variables are standardized. Shaded region = ±1 SD of gameful experience. Abbreviations: IU, Intolerance of Uncertainty; GAMEX, Gameful Experience; EI, Exercise Identity; SD, standard deviation.

The estimated chain indirect effect increased from .034 (95% CI [.001, .072]) at −1 SD IU to .085 (95% CI [.054, .123]) at mean IU and .136 (95% CI [.080, .201]) at +1 SD. Path 2, which does not contain EI, remained approximately .043 across IU levels by model specification. Estimated coefficients are displayed in Fig 4; they should be interpreted as conditional cross-sectional associations.

**Fig 4:**
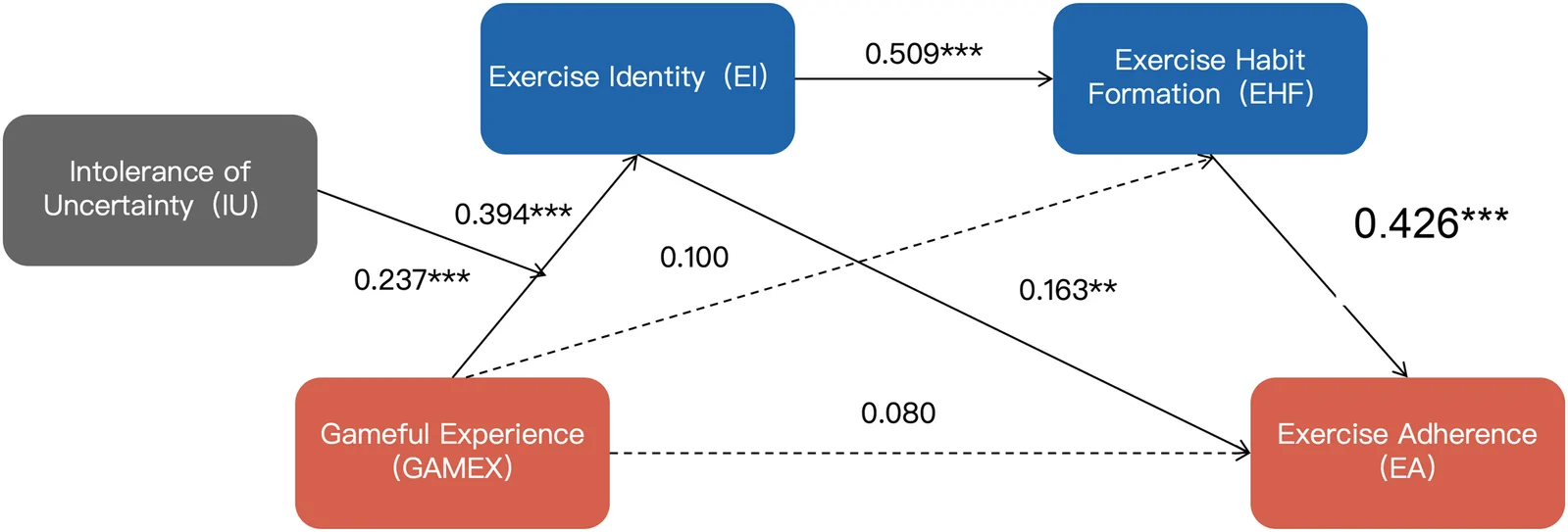
Path coefficient diagram with standardized estimates. Values on arrows are standardized regression coefficients (β). Dashed arrows indicate non-significant coefficients. ***p < .001, **p < .01. Abbreviations: GAMEX, Gameful Experience (X); EI, Exercise Identity (M1); EHF, Exercise Habit Formation (M2); IU, Intolerance of Uncertainty (W); EA, Exercise Adherence (Y).

## Discussion

### Gameful experience and exercise adherence

Gameful experience was positively associated with exercise adherence (β = .264, *p* < .001), consistent with H1. This result agrees with studies linking enjoyment and perceived competence to continued use of gamified exercise tools [5,36] and with Sun et al.’s finding that gamification-feature richness was associated with adherence intention through basic psychological needs [20]. The comparison is not uniformly supportive, however. Several trials found that gamified apps improved engagement or physical activity, whereas effects on goal adherence and maintenance were inconsistent [3,4,6]. Differences in follow-up duration, app features, behavioral outcomes, and the distinction between subjective gameful experience and assigned gamification may account for this mixed evidence. The present result supports an association between the quality of users’ gameful experience and adherence, rather than the stronger claim that adding game elements will necessarily sustain exercise. The GAMEX–EA coefficient was no longer significant after EI and EHF were entered, a pattern compatible with, but not proof of, the proposed indirect sequence.

### The mediating role of exercise identity

The GAMEX → EI → EA indirect estimate was .065 (95% CI [.020, .114]), supporting H2 at the associational level. Achievement feedback and visible progress may help users interpret repeated activity as evidence that exercise is part of who they are. This interpretation is consistent with identity-focused physical-activity app design [7,11], Ji et al.’s four-wave finding that exercise identity predicted later activity more consistently than the reverse path [8], and trial evidence linking post-intervention identity with maintenance [10]. In contrast, Schumacher et al. [12] found that changes in identity and accelerometer-measured exercise did not track one another reliably during weight-loss treatment. The discrepancy may reflect differences between university app users and adults in weight-loss treatment, between cross-sectional self-report and objective activity measures, or between stable identity differences and within-person identity change. Exercise identity may therefore be associated with maintenance, but the size and direction of the relation may depend on context and measurement.

The conditional indirect estimate varied with IU, as discussed below. Identity-reinforcing features such as cumulative achievement displays are therefore candidates for experimental testing, not established prescriptions for app design.

### The mediating role of exercise habit formation

The GAMEX → EHF → EA indirect estimate was .043 (95% CI [.002, .087]), supporting H3, although the lower confidence bound was close to zero. The conditional GAMEX–EHF coefficient was not significant (β = .100, *p* = .055). Together with the stronger EI–EHF association, this pattern is consistent with an identity-to-habit ordering, but reverse or reciprocal relations cannot be excluded. Longitudinal habit research and dual-process accounts provide theoretical support for the proposed order [13,17,18], while the micro-randomized trial by Baretta et al. [37] highlights the importance of contextual cues and commitment in app-supported habit formation.

Path 2 (GAMEX → EHF → EA) remained approximately .043 across IU levels because IU was specified to moderate only the GAMEX–EI path. This model-based stability should not be interpreted as evidence that habit processes are universal or independent of personality. Future studies could test whether contextual stability, implementation intentions, or specific app cues modify the GAMEX–habit association.

### The chain mediation pathway

The estimated chain indirect association (GAMEX → EI → EHF → EA) was .085 (95% CI [.054, .123]), supporting H4 statistically. The ordering is consistent with the Multi-Process Action Control framework, in which identity is related to reflective and automatic processes [18]. However, the cross-sectional estimate does not demonstrate that identity preceded habit or that either construct caused later adherence.

Recent studies among Chinese university students have also placed exercise identity and habit within multivariable indirect-effect models [38,39]. Those studies examined different outcomes and do not verify the ordering tested here, but they show that identity and repeated exercise behavior are embedded in broader motivational systems. The present chain estimate adds evidence that EI and EHF covary in the theoretically proposed order, while longitudinal comparisons of alternative orders remain necessary.

### The moderating role of intolerance of uncertainty

We examined IU as a possible boundary condition of the GAMEX–EI association (β = .237, *p* < .001, $\Delta R^{2}$ = .036). Individuals higher in IU showed a stronger association between gameful experience and exercise identity. This preliminary result requires replication; it does not demonstrate causal moderation.

The proposed interpretation draws on the information-seeking component of IU. In an experiment, participants higher in prospective IU sought more feedback when uncertain outcomes were personally relevant [23]. Progress bars, streaks, and achievement records may play a comparable uncertainty-reduction role by making exercise progress easier to monitor. This account is consistent with evidence that IU is linked to the emotional costs of uncertainty [24,40], but the analogy remains indirect because the present study measured overall gameful experience rather than exposure to particular feedback features. An alternative explanation is that highly engaged app users experience both greater certainty and stronger exercise identity, or that established exerciser identities make progress feedback more meaningful. These alternatives require experimental or longitudinal tests.

The estimated chain indirect effect differed across IU levels, but this observational result is not a product-design rule. Future experiments could manipulate predictable progress feedback and uncertain rewards separately, then test whether IU changes their effects on exercise identity and objectively measured adherence.

### Limitations and future directions

Several limitations should be acknowledged. First, the cross-sectional design prevents causal inference regarding the proposed sequence, and cross-sectional estimates of longitudinal mediation can be biased [41]. Future research should measure GAMEX, identity, automaticity, and adherence at separate waves and compare the proposed order with reverse and reciprocal models [8,10,12]. Randomized studies could manipulate gamified versus non-gamified feedback and follow behavior after the intervention to test causal effects and maintenance.

Second, all variables were self-reported online at one time point. Harman’s first factor accounted for 41.08%, but this weak diagnostic cannot exclude common method bias [33]. Momentary mood, acquiescence, inattentive responding, and other response styles may have affected several scales simultaneously. Future studies should use temporal separation, marker variables or a common latent factor, and objective indicators such as app logs or accelerometer data [3].

Third, the sample was restricted to Chinese university students, and chronological age was not collected. This omission limits sample characterization and generalizability beyond the represented academic-year groups. Cross-cultural and multi-population replication should record age and other relevant demographic variables.

Fourth, this study examined IU as the sole moderator. Future research might explore additional boundary conditions—such as exercise self-efficacy, autonomous motivation, or specific app design features—that may further qualify the chain mediation pathways identified here.

Fifth, no independent pilot study was conducted before the main survey. Although the translated measures showed high internal consistency and the hypothesized five-factor model fit the main-sample data better than competing models, these results are not a substitute for pretesting item comprehension and cultural equivalence in a separate sample. Future studies should use cognitive interviews and an independent pilot sample before fielding the full survey.

## Conclusions

This study examined whether gameful experience was associated with exercise adherence through exercise identity and habit formation and whether IU qualified the GAMEX–EI association among Chinese university students. In the sample of 300 students, gameful experience was positively associated with exercise adherence (β = .264, *p* < .001). The data were consistent with indirect associations through exercise identity (effect = .065, 95% CI [.020, .114]), habit formation (effect = .043, 95% CI [.002, .087]), and their hypothesized sequence (effect = .085, 95% CI [.054, .123]). IU moderated the GAMEX–EI association (β = .237, *p* < .001), and the estimated chain indirect effect was larger at higher IU. Because all variables were measured cross-sectionally by online self-report, these findings do not establish causality or temporal ordering, and alternative models remain plausible. If replicated in longitudinal or experimental research, the findings could inform the testing of app features that make progress visible, reinforce an exerciser identity, and support repetition in stable contexts. IU-sensitive personalization should remain an experimental design option rather than a recommendation for implementation until its effects on objectively measured adherence have been established.

## Supporting information

S1 Data Participant-level survey dataset and variable dictionary. The workbook contains the anonymized item-level responses for the 300 analyzed participants, derived scale scores, descriptive statistics, correlations, and variable coding.
(XLSX)

S1 File Survey on exercise-app experience and exercise behavior. English-language version of the questionnaire, including background questions, response scales, and scoring notes.
(DOCX)
