# Supplementary material for: Gameful experience, exercise identity, and exercise adherence among Chinese university students: A moderated chain mediation model
Source: PLoS One. 2026 Oct 6;21(10):e0353100. doi: 10.1371/journal.pone.0353100 (PMC13641342; doi:10.1371/journal.pone.0353100)
Supplement: Supplementary file 1 [file pone.0353100.s005.pdf]

Response to Reviewer

Manuscript PONE-D-26-28180R1

Gameful experience, exercise identity, and exercise adherence among Chinese university students: a moderated chain mediation model

Dear Dr. Karimi and Reviewer #2,

Thank you for the careful second-round review and for recognizing the improvement made in the previous revision. We appreciate the opportunity to address the remaining presentation and reporting issues. We have revised each point and describe the changes below. Page and figure numbering refer to the clean revised manuscript.

Response to Reviewer #2

Comment 1 — Dashes in the Abstract background

> ***Reviewer comment:*** *The background section of the Abstract contains too many dashes; please remove them.*

Response: Thank you for identifying this stylistic issue. We removed the parenthetical dash construction from the Abstract background. The definition of gameful experience is now incorporated with a conventional comma construction, which improves readability without changing the meaning. See the Abstract, Background.

Comment 2 — Expansion and organization of the Introduction

> ***Reviewer comment:*** *The Introduction should be expanded and divided into three independent paragraphs.*

Response: We agree. The Introduction has been expanded and reorganized into three substantive paragraphs before the hypotheses. Paragraph 1 establishes the physical-activity problem, reviews evidence on gamified fitness applications, and identifies the gap concerning sustained exercise and subjective gameful experience. Paragraph 2 develops the exercise-identity and habit-formation rationale and explains their proposed sequence. Paragraph 3 develops IU as a boundary condition, presents a competing explanation, describes the Chinese university context, and states the study model. The revised structure provides a clearer progression from background to the specific research question.

Comment 3 — Gender information in Fig 1 or Table 1

> ***Reviewer comment:*** *Gender-related sample information is still missing from Fig 1 or Table 1.*

Response: Thank you. Although gender counts were reported in the Participants subsection, we agree that they should also be visible in the requested display. The analytic-sample box in Fig 1 now reports 148 men (49.3%) and 152 women (50.7%), and the same information is repeated once in the Fig 1 caption.

Comment 4 — Duplicate title or caption in Fig 1

> ***Reviewer comment:*** *Fig 1 contains two sets of titles/captions and this should be corrected.*

Response: We agree. Fig 1 now has one journal-formatted caption only. Descriptive information about the participant flow and gender distribution has been consolidated into that single caption, and no separate or duplicate figure title is retained.

Comment 5 — Abbreviations in figure captions

> ***Reviewer comment:*** *If abbreviations appear in a figure caption, please use "abbreviations" to explain their full forms.*

Response: Thank you. Every caption containing shortened terms now includes a clearly labeled "Abbreviations:" statement. The captions define GAMEX, EI, EHF, IU, EA, and, where relevant, SD. The format has been applied consistently to Figs 1–4.

Comment 6 — Size and clarity of Figs 2–4

> ***Reviewer comment:*** *Please enlarge Figs 2, 3, and 4, including all text inside and outside the figures.*

Response: We agree. Figs 2–4 are now displayed at 98% of the manuscript text width. For Figs 2 and 4, unused white margins were removed so that the model boxes, path labels, and coefficients occupy substantially more of the available width. Their TIFF files are RGB, LZW-compressed, 600 dpi, and 3274 × 1222 pixels. Fig 3 is supplied as an RGB TIFF at 300 dpi and 1884 × 1282 pixels and is also enlarged in the manuscript. We additionally corrected the EHF–EA significance marker in Fig 4 to 0.426*** so that it matches Table 5 ($p < .001$).

Comment 7 — Explicit comparison with published studies

> ***Reviewer comment:*** *Discuss the results in relation to published research and state clearly whether the findings agree or conflict with existing evidence.*

Response: Thank you. We made the comparisons more explicit in the Discussion. The GAMEX–adherence result is now described as agreeing with studies that link enjoyment, competence, and gamification-feature richness with continued use or adherence intention. We then contrast it with trials reporting inconsistent maintenance effects and discuss differences in follow-up duration, app features, outcomes, and assigned gamification versus subjective gameful experience. The exercise-identity section now identifies both agreement with longitudinal and intervention evidence and disagreement with evidence from behavioral weight-loss treatment, followed by possible contextual and measurement explanations. These revisions appear in "Gameful experience and exercise adherence" and "The mediating role of exercise identity."

Comment 8 — Aim and practical implications in the Conclusion

> ***Reviewer comment:*** *The Conclusion should begin with a brief statement of the study aim and end with potential practical applications.*

Response: We agree. The Conclusion now opens by stating that the study examined indirect associations of gameful experience with adherence through exercise identity and habit formation and tested IU as a qualifier of the GAMEX–EI association. It ends with a cautious practical implication: if the pattern is replicated longitudinally or experimentally, researchers and developers could test progress-visibility, identity-supportive, and stable-context features. We retain the important qualification that IU-sensitive personalization is an experimental option rather than an established implementation recommendation.

Closing

We thank the reviewer again for these precise suggestions. They improved the organization of the Introduction, the transparency of the sample description, the readability and consistency of the figures, and the balance of the Discussion and Conclusion. We hope that the revised manuscript now addresses all remaining concerns.

Sincerely,
Jingjing Song, Jiaying Li, and Dan Li
