# Supplementary material for: Gameful experience, exercise identity, and exercise adherence among Chinese university students: A moderated chain mediation model
Source: PLoS One. 2026 Oct 6;21(10):e0353100. doi: 10.1371/journal.pone.0353100 (PMC13641342; doi:10.1371/journal.pone.0353100)
Supplement: Supplementary file 2 [file pone.0353100.s002.docx]

**Survey on Exercise App Experience and Exercise Behavior**

**Dear Participant,** Thank you for taking the time to complete this survey. This questionnaire aims to investigate your experience using exercise-related mobile applications (apps) and your associated exercise behaviors. All data collected will be used solely for academic research purposes, kept strictly confidential, and will in no way affect you adversely. Please answer each question honestly and independently based on your own experience. There are no right or wrong answers. **Your participation is greatly appreciated and essential to this research.**

**Part 1 Background Information**

| **Gender** | □ Male □ Female |
| --- | --- |
| **Year of Study** | □ Year 1 □ Year 2 □ Year 3 □ Year 4 or above |
| **App Name** | (Please specify) ___________________________________ |
| **Duration of Use** | □ < 3 months □ 3–6 months □ 6–12 months □ > 1 year |
| **Weekly Frequency** | □ < 1 time □ 1–2 times □ 3–4 times □ ≥ 5 times |

**Part 2 Gameful Experience (GAMEX)**

*The following questions ask about your subjective experience while using the exercise app named above. Please circle the number that best reflects your actual experience.*

**Scoring:** 1 = Strongly Disagree	2 = Disagree	3 = Slightly Disagree	4 = Neutral	5 = Slightly Agree	6 = Agree	7 = Strongly Agree

**Section 1 — Enjoyment**

| **No.** | **Item** | **1** | **2** | **3** | **4** | **5** | **6** | **7** |
| --- | --- | --- | --- | --- | --- | --- | --- | --- |
| 1 | Using this exercise app is a lot of fun. | ○ | ○ | ○ | ○ | ○ | ○ | ○ |
| 2 | I like using this exercise app. | ○ | ○ | ○ | ○ | ○ | ○ | ○ |
| 3 | I thoroughly enjoy the process of using this exercise app. | ○ | ○ | ○ | ○ | ○ | ○ | ○ |
| 4 | The experience of using this exercise app is pleasant. | ○ | ○ | ○ | ○ | ○ | ○ | ○ |
| 5 | I find this exercise app very entertaining. | ○ | ○ | ○ | ○ | ○ | ○ | ○ |
| 6 | Even without any requirement, I would voluntarily use this exercise app. | ○ | ○ | ○ | ○ | ○ | ○ | ○ |

**Section 2 — Absorption**

| **No.** | **Item** | **1** | **2** | **3** | **4** | **5** | **6** | **7** |
| --- | --- | --- | --- | --- | --- | --- | --- | --- |
| 7 | When using this app, I forget where I am. | ○ | ○ | ○ | ○ | ○ | ○ | ○ |
| 8 | When using this app, I lose awareness of my surroundings. | ○ | ○ | ○ | ○ | ○ | ○ | ○ |
| 9 | After using this app, I feel as if I have returned from another world. | ○ | ○ | ○ | ○ | ○ | ○ | ○ |
| 10 | Using this app makes me temporarily forget all the worries in my life. | ○ | ○ | ○ | ○ | ○ | ○ | ○ |
| 11 | When using this app, I am completely unaware of everything around me. | ○ | ○ | ○ | ○ | ○ | ○ | ○ |
| 12 | When using this app, I completely lose track of time. | ○ | ○ | ○ | ○ | ○ | ○ | ○ |

**Section 3 — Creative Thinking**

| **No.** | **Item** | **1** | **2** | **3** | **4** | **5** | **6** | **7** |
| --- | --- | --- | --- | --- | --- | --- | --- | --- |
| 13 | Using this app stimulates my imagination. | ○ | ○ | ○ | ○ | ○ | ○ | ○ |
| 14 | When using this app, I feel full of creativity. | ○ | ○ | ○ | ○ | ○ | ○ | ○ |
| 15 | When using this app, I feel I can explore many new things. | ○ | ○ | ○ | ○ | ○ | ○ | ○ |
| 16 | When using this app, I feel a sense of adventure. | ○ | ○ | ○ | ○ | ○ | ○ | ○ |

**Section 4 — Activation**

| **No.** | **Item** | **1** | **2** | **3** | **4** | **5** | **6** | **7** |
| --- | --- | --- | --- | --- | --- | --- | --- | --- |
| 17 | When using this app, I feel fully energized. | ○ | ○ | ○ | ○ | ○ | ○ | ○ |
| 18 | When using this app, I feel excited and eager to take action. | ○ | ○ | ○ | ○ | ○ | ○ | ○ |
| 19 | When using this app, I feel thrilled and stimulated. | ○ | ○ | ○ | ○ | ○ | ○ | ○ |
| 20 | When using this app, I feel highly aroused and full of vitality. | ○ | ○ | ○ | ○ | ○ | ○ | ○ |

**Section 5 — Absence of Negative Affect**

| **No.** | **Item** | **1** | **2** | **3** | **4** | **5** | **6** | **7** |
| --- | --- | --- | --- | --- | --- | --- | --- | --- |
| 21 | When using this app, I feel irritated and unsettled. (R) | ○ | ○ | ○ | ○ | ○ | ○ | ○ |
| 22 | When using this app, I feel bored and disgusted. (R) | ○ | ○ | ○ | ○ | ○ | ○ | ○ |
| 23 | When using this app, I feel dejected and discouraged. (R) | ○ | ○ | ○ | ○ | ○ | ○ | ○ |

***Note.*** *Items marked "(R)" are reverse-scored during data analysis (original 7 → 1, 1 → 7).*

**Section 6 — Dominance**

| **No.** | **Item** | **1** | **2** | **3** | **4** | **5** | **6** | **7** |
| --- | --- | --- | --- | --- | --- | --- | --- | --- |
| 24 | When using this app, I feel in complete control and at ease. | ○ | ○ | ○ | ○ | ○ | ○ | ○ |
| 25 | When using this app, I feel influential. | ○ | ○ | ○ | ○ | ○ | ○ | ○ |
| 26 | When using this app, I feel fully autonomous in making decisions. | ○ | ○ | ○ | ○ | ○ | ○ | ○ |
| 27 | When using this app, I feel confident and self-assured. | ○ | ○ | ○ | ○ | ○ | ○ | ○ |

**Part 3 Exercise Identity (EIS)**

*The following questions ask about your personal identification with exercise. Please answer based on your genuine thoughts.*

**Scoring:** 1 = Strongly Disagree	2 = Disagree	3 = Slightly Disagree	4 = Neutral	5 = Slightly Agree	6 = Agree	7 = Strongly Agree

| **No.** | **Item** | **1** | **2** | **3** | **4** | **5** | **6** | **7** |
| --- | --- | --- | --- | --- | --- | --- | --- | --- |
| 28 | I consider myself to be a person who exercises regularly. | ○ | ○ | ○ | ○ | ○ | ○ | ○ |
| 29 | When introducing myself to others, I usually mention that I exercise regularly. | ○ | ○ | ○ | ○ | ○ | ○ | ○ |
| 30 | I have a lot in common with people who enjoy being physically active. | ○ | ○ | ○ | ○ | ○ | ○ | ○ |
| 31 | Exercise / physical activity is a central aspect of how I see myself. | ○ | ○ | ○ | ○ | ○ | ○ | ○ |
| 32 | I need to exercise to feel good about myself. | ○ | ○ | ○ | ○ | ○ | ○ | ○ |
| 33 | Others see me as someone who has a regular exercise habit. | ○ | ○ | ○ | ○ | ○ | ○ | ○ |
| 34 | For me, being an exerciser means more than just working out—it represents an active and positive lifestyle. | ○ | ○ | ○ | ○ | ○ | ○ | ○ |
| 35 | If I were forced to give up exercise, I would feel a real sense of loss. | ○ | ○ | ○ | ○ | ○ | ○ | ○ |
| 36 | Exercise is an indispensable and central part of my life. | ○ | ○ | ○ | ○ | ○ | ○ | ○ |

**Part 4 Exercise Habit Formation (SRBAI)**

*The following questions ask about the degree of automaticity in your current exercise behavior. Please answer based on your actual daily habits.*

**Scoring:** 1 = Strongly Disagree	2 = Disagree	3 = Slightly Disagree	4 = Neutral	5 = Slightly Agree	6 = Agree	7 = Strongly Agree

**Stem:** *For me, doing exercise is something that…*

| **No.** | **Item** | **1** | **2** | **3** | **4** | **5** | **6** | **7** |
| --- | --- | --- | --- | --- | --- | --- | --- | --- |
| 37 | …I do automatically. | ○ | ○ | ○ | ○ | ○ | ○ | ○ |
| 38 | …I do without having to consciously remember to do. | ○ | ○ | ○ | ○ | ○ | ○ | ○ |
| 39 | …I do without thinking. | ○ | ○ | ○ | ○ | ○ | ○ | ○ |
| 40 | …I start doing before I realize I am doing it. | ○ | ○ | ○ | ○ | ○ | ○ | ○ |

**Part 5 Intolerance of Uncertainty (IUS-12)**

*The following questions ask about your typical feelings and reactions when facing uncertain situations. Please answer based on how you generally feel.*

**Scoring:** 1 = Not at all characteristic of me	2 = A little characteristic	3 = Somewhat characteristic	4 = Very characteristic	5 = Entirely characteristic of me

**Subscale A — Prospective Anxiety (IUS-P)**

| **No.** | **Item** | **1** | **2** | **3** | **4** | **5** |
| --- | --- | --- | --- | --- | --- | --- |
| 41 | Unforeseen events upset me greatly. | ○ | ○ | ○ | ○ | ○ |
| 42 | It frustrates me not having all the information I need. | ○ | ○ | ○ | ○ | ○ |
| 43 | Uncertainty keeps me from living a full life. | ○ | ○ | ○ | ○ | ○ |
| 44 | I always want to be well-prepared so that nothing catches me off guard. | ○ | ○ | ○ | ○ | ○ |
| 45 | Even a minor, unexpected event can ruin my entire plans. | ○ | ○ | ○ | ○ | ○ |
| 46 | When it is time to act, uncertainty paralyzes me. | ○ | ○ | ○ | ○ | ○ |
| 47 | When I am uncertain, I cannot function well. | ○ | ○ | ○ | ○ | ○ |

**Subscale B — Inhibitory Anxiety (IUS-I)**

| **No.** | **Item** | **1** | **2** | **3** | **4** | **5** |
| --- | --- | --- | --- | --- | --- | --- |
| 48 | I always want to know what the future holds for me. | ○ | ○ | ○ | ○ | ○ |
| 49 | I cannot stand being taken by surprise. | ○ | ○ | ○ | ○ | ○ |
| 50 | The slightest doubt can stop me from taking action. | ○ | ○ | ○ | ○ | ○ |
| 51 | I must be able to plan everything in advance before acting. | ○ | ○ | ○ | ○ | ○ |
| 52 | I must get away from all uncertain situations. | ○ | ○ | ○ | ○ | ○ |

***Note.*** *Higher scores on this scale indicate greater intolerance of uncertainty.*

**Part 6 Exercise Adherence (Adapted from EARS)**

*The following questions ask about your actual exercise adherence over the past month. Please answer based on your true situation.*

**Scoring:** 1 = Not at all true	2 = Slightly true	3 = Moderately true	4 = Mostly true	5 = Completely true

| **No.** | **Item** | **1** | **2** | **3** | **4** | **5** |
| --- | --- | --- | --- | --- | --- | --- |
| 53 | I am able to maintain exercise at the frequency I set for myself. | ○ | ○ | ○ | ○ | ○ |
| 54 | Even when I feel tired or pressed for time, I still complete my exercise plan. | ○ | ○ | ○ | ○ | ○ |
| 55 | The amount of exercise I actually do meets the targets I set for myself. | ○ | ○ | ○ | ○ | ○ |
| 56 | When I encounter obstacles during exercise, I stop and give up. (R) | ○ | ○ | ○ | ○ | ○ |
| 57 | I flexibly adjust my exercise approach to make sure I can keep it up. | ○ | ○ | ○ | ○ | ○ |
| 58 | I find it difficult to incorporate exercise into my daily routine. (R) | ○ | ○ | ○ | ○ | ○ |

***Note.*** *Items marked "(R)" are reverse-scored during data entry (5→1, 4→2, 3→3, 2→4, 1→5).*

***— Thank you for your patience and participation. The survey is now complete. —***

**Part	Appendix: Scale Overview**

| **Survey Part** | **Variable** | **Scale Source** | **Items** | **Scoring** |
| --- | --- | --- | --- | --- |
| Part 2 | Gameful Experience (Independent Variable) | GAMEX (Eppmann et al., 2018) | 27 | 7-point; items 21–23 reverse-scored |
| Part 3 | Exercise Identity (Mediator 1) | EIS (Anderson & Cychosz, 1994) | 9 | 7-point; all forward-scored |
| Part 4 | Exercise Habit Formation (Mediator 2) | SRBAI (Gardner et al., 2012) | 4 | 7-point; all forward-scored |
| Part 5 | Intolerance of Uncertainty (Moderator) | IUS-12 (Carleton et al., 2007; Wu et al., 2016) | 12 | 5-point; all forward-scored |
| Part 6 | Exercise Adherence (Outcome) | Adapted from EARS (Newman-Beinart et al., 2017) | 6 | 5-point; items 56 & 58 reverse-scored |
| **Total** |  |  | **58** |  |
