# Supplementary material for: Gameful experience, exercise identity, and exercise adherence among Chinese university students: A moderated chain mediation model
Source: PLoS One. 2026 Oct 6;21(10):e0353100. doi: 10.1371/journal.pone.0353100 (PMC13641342; doi:10.1371/journal.pone.0353100)
Supplement: Peer review history

# Peer review: Decision Letter 0

**DOI:** 10.1371/journal.pone.0353100.r001

**Authors:** Amir Karimi

4 Aug 2026

Dear Dr. Song,

Thank you for submitting your manuscript to PLOS ONE. After careful consideration, we feel that it has merit but does not fully meet PLOS ONE’s publication criteria as it currently stands. Therefore, we invite you to submit a revised version of the manuscript that addresses the points raised during the review process.

Please submit your revised manuscript by Oct 03 2026 11:59PM. If you will need more time than this to complete your revisions, please reply to this message or contact the journal office at plosone@plos.org. When you're ready to submit your revision, log on to https://www.editorialmanager.com/pone/ and select the 'Submissions Needing Revision' folder to locate your manuscript file.

- A letter that responds to each point raised by the academic editor and reviewer(s). You should upload this letter as a separate file labeled 'Response to Reviewers'.
- A marked-up copy of your manuscript that highlights changes made to the original version. You should upload this as a separate file labeled 'Revised Manuscript with Track Changes'.
- An unmarked version of your revised paper without tracked changes. You should upload this as a separate file labeled 'Manuscript'.

If you would like to make changes to your financial disclosure, please include your updated statement in your cover letter. Guidelines for resubmitting your figure files are available below the reviewer comments at the end of this letter.

If applicable, we recommend that you deposit your laboratory protocols in protocols.io to enhance the reproducibility of your results. Protocols.io assigns your protocol its own identifier (DOI) so that it can be cited independently in the future. For instructions see: https://journals.plos.org/plosone/s/submission-guidelines#loc-laboratory-protocols. Additionally, PLOS ONE offers an option for publishing peer-reviewed Lab Protocol articles, which describe protocols hosted on protocols.io. Read more information on sharing protocols at https://plos.org/protocols?utm_medium=editorial-email&utm_source=authorletters&utm_campaign=protocols.

As the corresponding author, your ORCID iD is verified in the submission system and will appear in the published article. PLOS supports the use of ORCID, and we encourage all coauthors to register for an ORCID iD and use it as well. Please encourage your coauthors to verify their ORCID iD within the submission system before final acceptance, as unverified ORCID iDs will not appear in the published article. *Only*  the individual author can complete the verification step; PLOS staff *cannot*  verify ORCID iDs on behalf of authors.

We look forward to receiving your revised manuscript.

Kind regards,

Amir Karimi, PhD

Academic Editor

PLOS One

Journal Requirements:

When submitting your revision, we need you to address these additional requirements.

1. Please ensure that your manuscript meets PLOS ONE's style requirements, including those for file naming. The PLOS ONE style templates can be found at

https://journals.plos.org/plosone/s/file?id=wjVg/PLOSOne_formatting_sample_main_body.pdf and https://journals.plos.org/plosone/s/file?id=ba62/PLOSOne_formatting_sample_title_authors_affiliations.pdf

2. Please include captions for your Supporting Information files at the end of your manuscript, and update any in-text citations to match accordingly. Please see our Supporting Information guidelines for more information: http://journals.plos.org/plosone/s/supporting-information.

3. If the reviewer comments include a recommendation to cite specific previously published works, please review and evaluate these publications to determine whether they are relevant and should be cited. There is no requirement to cite these works unless the editor has indicated otherwise.

[Note: HTML markup is below. Please do not edit.]

Reviewers' comments:

Reviewer's Responses to Questions

**Comments to the Author**

1. Is the manuscript technically sound, and do the data support the conclusions?

Reviewer #1: Yes

Reviewer #2: Partly

**********

2. Has the statistical analysis been performed appropriately and rigorously?

Reviewer #1: Yes

Reviewer #2: Yes

**********

3. Have the authors made all data underlying the findings in their manuscript fully available?

The PLOS Data policy

Reviewer #1: No

Reviewer #2: Yes

**********

4. Is the manuscript presented in an intelligible fashion and written in standard English?

Reviewer #1: Yes

Reviewer #2: No

**********

Reviewer #1: My primary concern relates to the interpretation of the findings. Although the authors acknowledge that the study employs a cross-sectional design, the manuscript repeatedly presents the proposed relationships as revealing a sequential psychological mechanism or psychological architecture through which gameful experience promotes exercise adherence. Such causal and temporal interpretations are not warranted from cross-sectional self-report data. The mediation model is statistically plausible, but alternative causal orderings are equally possible and cannot be ruled out. Throughout the Discussion and Conclusion, the language should therefore be revised to emphasize associations that are theoretically consistent with the proposed model rather than demonstrated causal processes.

A second concern involves the theoretical justification for the moderating role of intolerance of uncertainty. The inclusion of this construct is potentially interesting and may represent an original aspect of the study; however, the theoretical rationale remains relatively speculative. The argument that gamified applications provide "structured certainty" for individuals with higher intolerance of uncertainty is intriguing but requires stronger conceptual support from the existing literature. Expanding the theoretical discussion and acknowledging competing explanations would substantially strengthen the manuscript.

I also encourage the authors to moderate the claims regarding novelty and practical implications. Several statements describing the findings as revealing a new psychological architecture or providing the first empirical demonstration appear stronger than the evidence supports. Likewise, the recommendations for designing gamified applications that personalize interventions based on intolerance of uncertainty extend beyond what can be concluded from an observational survey. Presenting these implications as promising directions rather than established design principles would improve the scientific balance of the paper.

From a methodological perspective, all variables were collected using self-report questionnaires administered at a single time point, creating the potential for common method variance. Although Harman's single-factor test was reported, this approach is widely recognized as a relatively weak diagnostic. If additional analyses are not feasible, the authors should provide a more balanced discussion of this limitation and acknowledge that common method bias cannot be excluded. Furthermore, a brief justification for the use of item parceling in the confirmatory factor analysis and the selection of PROCESS regression rather than structural equation modeling for hypothesis testing would improve methodological transparency.

Finally, the manuscript could benefit from some editing to improve conciseness. Several findings are repeated across the Results, Discussion, and Conclusion with very similar wording. Reducing repetition and focusing the Discussion more explicitly on the broader theoretical implications and limitations would enhance readability.

Reviewer #2: Manuscript Number: PONE-D-26-28180

Despite the interesting topic, there are certain concerns that should be addressed before further consideration.

1.The manuscript requires thorough linguistic revision. Many sentences are overly long, and some text appears to rely on AI-generated content.

2.The introduction fails to adequately establish the research background, the limitations of existing studies, or the specific research gap being addressed

3.The study’s novelty regarding the impact of gameful experience (GAMEX) on EI, EHF, IU, and EA must be clearly articulated.

4.Ensure consistent use of full terms and their corresponding abbreviations throughout the manuscript.

5.H5 should be revised to be consistent with the study.

6.The age of the population is missing from the method section.

7.The participants and data collection sections lack information regarding eligibility criteria and participants' withdrawal.

8.Was the validity of the Chinese translated questionnaires examined? Was a pilot study conducted prior to the main study?

9.What are the names of the Chinese Apps used in this study?

10.Data for all variables were collected online; does this reflect differences in student mood and response style?

11.A flow diagram is required to illustrate research activities and measurements. Are there differences between students based on gender, academic year, or duration of app usage (three months versus more than one year)?

12.Improve Fig. 1 by enlarging the text. Use either the full term or the abbreviation consistently in the figure caption.

13.Compare the results with findings from relevant published studies.

14.Address the data analysis of primary findings in the conclusion section.

15.Suggestions for future research should be addressed at the end of the conclusion section.

**********

what does this mean?). If published, this will include your full peer review and any attached files.

If you choose “no”, your identity will remain anonymous but your review may still be made public.

**Do you want your identity to be public for this peer review?**  For information about this choice, including consent withdrawal, please see our Privacy Policy

Reviewer #1: No

Reviewer #2: No

**********

[NOTE: If reviewer comments were submitted as an attachment file, they will be attached to this email and accessible via the submission site. Please log into your account, locate the manuscript record, and check for the action link "View Attachments". If this link does not appear, there are no attachment files.]

To ensure your figures meet our technical requirements, please review our figure guidelines: https://journals.plos.org/plosone/s/figures

You may also use PLOS’s free figure tool, NAAS, to help you prepare publication quality figures: https://journals.plos.org/plosone/s/figures#loc-tools-for-figure-preparation.

NAAS will assess whether your figures meet our technical requirements by comparing each figure against our figure specifications.

# Author comment: Author response to Decision Letter 1

**DOI:** 10.1371/journal.pone.0353100.r002

10 Aug 2026

Response to Reviewers

Manuscript PONE-D-26-28180 — “Gameful experience, exercise identity, and exercise adherence among Chinese university students: a moderated chain mediation model”

Dear Dr. Karimi, Reviewer #1, and Reviewer #2,

Thank you very much for the careful and constructive assessment of our manuscript. We appreciate the time and expertise invested by the Academic Editor and both reviewers. The comments helped us identify places where the original submission overstated what could be inferred from cross-sectional data, did not explain the theoretical role of intolerance of uncertainty (IU) sufficiently, and lacked important methodological and reporting details. We have revised the manuscript extensively and respond to every point below. Reviewer comments are reproduced in italics, followed by our response and the location of the corresponding revision in the marked manuscript.

For orientation, the principal changes are:

• Causal and temporal claims were replaced with association-based language throughout the Abstract, Results, Discussion, and Conclusions.

• The theoretical rationale for IU was expanded and now includes both certainty-seeking and competing distress/avoidance explanations.

• Ten relevant publications were added to strengthen the gamification, exercise-identity, IU, cross-sectional mediation, item-parceling, and PROCESS-versus-SEM arguments.

• Eligibility, voluntary withdrawal, app names, the absence of chronological-age data, and the absence of an independent pilot study are now reported explicitly.

• A participant-flow diagram and subgroup analyses by gender, academic year, and app-use duration were added.

• The Discussion now compares the findings with supportive and conflicting published evidence, and practical recommendations are framed as questions for future experiments.

Response to the Academic Editor — Journal Requirements

Requirement 1 — PLOS ONE style and file naming

Response: We reviewed the PLOS ONE formatting samples and retained the manuscript in the journal-compatible LaTeX format. We provide a clean manuscript, a marked manuscript, the bibliography file, and separate figure files with PLOS ONE-compatible names.

Requirement 2 — Supporting Information captions and in-text citations

Response: We added a Supporting Information captions section at the end of the manuscript. The anonymized participant-level workbook is designated S1 Data and the questionnaire is designated S1 File. The Data Availability statement and relevant in-text references were updated accordingly.

Requirement 3 — Evaluation of relevant previously published work

Response: Although neither reviewer required citation of a specific paper, both reviewers asked us to strengthen the theoretical rationale and compare our findings with prior studies. We therefore conducted a targeted literature review and added ten directly relevant sources. These include randomized trials and meta-analyses of gamified physical-activity interventions; supportive and conflicting longitudinal evidence on exercise identity; an experiment on IU and information seeking; and methodological sources on cross-sectional mediation, item parceling, and PROCESS versus SEM. We added only papers that informed a specific statement or comparison.

Response to Reviewer #1

We thank Reviewer #1 for a thoughtful and methodologically careful review. We agree that the original manuscript went beyond what a cross-sectional self-report design can support. The revisions prompted by these comments have made the manuscript more balanced and transparent.

R1, Comment 1 — Causal and temporal interpretation of the cross-sectional mediation model

“Although the authors acknowledge that the study employs a cross-sectional design, the manuscript repeatedly presents the proposed relationships as revealing a sequential psychological mechanism or psychological architecture through which gameful experience promotes exercise adherence. Such causal and temporal interpretations are not warranted from cross-sectional self-report data…”

Response: We agree and thank the reviewer for identifying the extent of this problem. We systematically revised the Abstract, Introduction, Results, Discussion, and Conclusions. Terms such as “reveals,” “promotes,” “psychological architecture,” and statements implying that identity necessarily precedes habit were removed or qualified. The revised manuscript now states that the observed associations are consistent with the hypothesized ordering, while reverse, reciprocal, and other equivalent models remain possible. We also added Maxwell and Cole (2007) to support the methodological warning that cross-sectional estimates of longitudinal mediation can be biased. See the Abstract Conclusions, the opening of Participants and methods, each Discussion subsection, and Limitations and future directions.

R1, Comment 2 — Theoretical justification for the moderating role of IU

“The argument that gamified applications provide “structured certainty” for individuals with higher intolerance of uncertainty is intriguing but requires stronger conceptual support from the existing literature. Expanding the theoretical discussion and acknowledging competing explanations would substantially strengthen the manuscript.”

Response: We appreciate this suggestion and agree that the original rationale was too speculative. The Introduction now distinguishes predictable feedback (progress bars, streaks, achievement records) from uncertain app elements (variable challenges and competitive outcomes). We added Bartoszek et al. (2022), whose experiment links prospective IU with feedback-seeking under personally relevant uncertainty, as direct support for the information-seeking component of our argument. We also added competing explanations: uncertain rewards may increase distress or avoidance, established exercise identity may make feedback more meaningful, and highly engaged users may experience both greater certainty and stronger identity. The Discussion now treats “structured certainty” as one interpretation to be tested, not as an established mechanism. See the IU paragraph in the Introduction and The moderating role of intolerance of uncertainty in the Discussion.

R1, Comment 3 — Novelty claims and practical implications

“Several statements describing the findings as revealing a new psychological architecture or providing the first empirical demonstration appear stronger than the evidence supports… Presenting these implications as promising directions rather than established design principles would improve the scientific balance of the paper.”

Response: We agree. Claims of a “new psychological architecture” and a “first empirical demonstration” were deleted. We now describe the contribution as testing a theoretically specified association pattern and examining IU as a possible boundary condition. Recommendations concerning identity-reinforcing or IU-personalized features are presented as candidates for longitudinal and experimental testing. See the final Introduction paragraph, The moderating role of intolerance of uncertainty, and Conclusions.

R1, Comment 4 — Common method variance and methodological transparency

“Although Harman’s single-factor test was reported, this approach is widely recognized as a relatively weak diagnostic… A brief justification for the use of item parceling in the confirmatory factor analysis and the selection of PROCESS regression rather than structural equation modeling would improve methodological transparency.”

Response: Thank you; we agree on all three points. First, the Results no longer interpret Harman’s test as showing that common method bias is absent. The revised Limitations section states that mood, acquiescence, inattentive responding, and other response styles may contribute to shared covariance. Second, the Data analysis subsection explains that parcels represent prespecified scale dimensions and were used only after internal consistency and dimensionality were examined; Little et al. (2002) is now cited. Third, we explain that PROCESS was chosen to estimate conditional indirect effects, interactions, simple slopes, and bootstrap intervals for observed composite scores. Hayes, Montoya, and Rockwood (2017) is cited for the equivalence of coefficient estimates between regression-based PROCESS and observed-variable SEM path models. See Common method bias and descriptive statistics, Data analysis, and Limitations and future directions.

R1, Comment 5 — Conciseness and repetition

“Several findings are repeated across the Results, Discussion, and Conclusion with very similar wording. Reducing repetition and focusing the Discussion more explicitly on the broader theoretical implications and limitations would enhance readability.”

Response: We agree and appreciate this editorial guidance. Repeated numerical descriptions and duplicated interpretations were removed. The Results now reports the estimates without extended theoretical commentary; the Discussion compares them with published evidence and explains uncertainty; the Conclusion gives only the principal estimates, interpretive boundary, and future directions. We also shortened long sentences throughout.

R1, Data availability assessment

“Have the authors made all data underlying the findings in their manuscript fully available? Reviewer #1: No.”

Response: We appreciate the opportunity to clarify this. We recognize that the Figshare record was not immediately accessible to the reviewer. We therefore provide the complete anonymized participant-level item responses, variable definitions, and derived scores as S1 Data, without restriction. A copy remains deposited in Figshare at https://doi.org/10.6084/m9.figshare.32584320. The revised Data Availability statement identifies S1 Data as the direct access route.

Response to Reviewer #2

We thank Reviewer #2 for the detailed review and for identifying several reporting omissions. We address the comments in the order presented.

R2, Comment 1 — English-language revision

“The manuscript requires thorough linguistic revision. Many sentences are overly long, and some text appears to rely on AI-generated content.”

Response: We agree that the prose required substantial revision. We edited the full manuscript for sentence length, repetition, terminology, and unsupported rhetorical claims. The Abstract, Introduction, Discussion, and Conclusion received the most extensive rewriting. We retained a transparent statement in the Acknowledgments describing the limited use of ChatGPT-4 for language editing and affirming that the authors reviewed and take responsibility for the content.

R2, Comment 2 — Background and research gap

“The introduction fails to adequately establish the research background, the limitations of existing studies, or the specific research gap being addressed.”

Response: Thank you for this comment. The Introduction now distinguishes objective gamification features from subjective gameful experience, summarizes randomized-trial and meta-analytic evidence, and explains that intervention effects vary across features and follow-up periods. It then identifies two specific gaps: limited evidence on continued exercise rather than short-term app use, and limited integration of exercise identity and habit automaticity in one model. See the first two paragraphs of the revised Introduction.

R2, Comment 3 — Contribution concerning GAMEX, EI, EHF, IU, and EA

“The study’s novelty regarding the impact of gameful experience (GAMEX) on EI, EHF, IU, and EA must be clearly articulated.”

Response: We appreciate the opportunity to clarify the contribution. Because IU is a moderator rather than an outcome, the revised manuscript does not describe GAMEX as having an “impact” on IU. The contribution is now framed as examining whether EI and EHF form a theoretically specified indirect association between GAMEX and EA and whether the GAMEX–EI association varies by IU. We also moderated claims of novelty to remain consistent with the observational design. See the final Introduction paragraph.

R2, Comment 4 — Consistent terminology and abbreviations

“Ensure consistent use of full terms and their corresponding abbreviations throughout the manuscript.”

Response: We agree. Each construct is introduced with its full name before the abbreviation, and GAMEX, EI, EHF, IU, and EA are now used consistently in the text, tables, figures, and captions.

R2, Comment 5 — Revision of H5

“H5 should be revised to be consistent with the study.”

Response: Thank you. H5 now states that the positive association between GAMEX and EI varies as a function of IU and is stronger at higher levels of IU. This wording matches the tested cross-sectional interaction and avoids implying that the study established a causal first-stage effect.

R2, Comment 6 — Participant age

“The age of the population is missing from the method section.”

Response: We agree that age would normally be reported. Chronological age was not collected, so an age mean or range cannot be reconstructed without inventing data. We now state this omission explicitly in Participants and data collection and in Limitations. Academic-year distributions are reported as the available description of educational stage.

R2, Comment 7 — Eligibility criteria and participant withdrawal

“The participants and data collection sections lack information regarding eligibility criteria and participants’ withdrawal.”

Response: Thank you for noting this omission. We added the eligibility criteria: current university enrollment, current use of an exercise app with game-like features, and informed consent. We also clarified the online withdrawal procedure. Participants could leave by closing the questionnaire before final submission; Questionnaire Star did not upload these incomplete responses, and they were not retained in the analytic dataset. The manuscript distinguishes such non-submissions from the 320 completed questionnaires and the 20 completed responses later excluded during data cleaning. See Participants and data collection and Fig 1.

R2, Comment 8 — Chinese questionnaires and pilot study

“Was the validity of the Chinese translated questionnaires examined? Was a pilot study conducted prior to the main study?”

Response: We appreciate this important question. We now report the translation and back-translation procedure and the main-sample psychometric evidence, including Cronbach’s alpha, CFA model comparisons, and standardized factor loadings. No independent pilot study was conducted before the main survey, and we state this directly. We do not present a retrospective subsample of the main data as a pilot because that would not constitute independent pretesting. The Limitations section now explains that main-sample reliability and CFA do not replace cognitive interviewing or independent pretesting of item comprehension and cultural equivalence. See the end of Measures and the new fifth limitation.

R2, Comment 9 — Names of Chinese apps

“What are the names of the Chinese Apps used in this study?”

Response: Thank you. We now report the app distribution: Keep (n = 94), Codoon/Gu Dong (n = 77), Yuedongquan (n = 67), Nike (n = 37), and other apps (n = 25). See Participants and data collection.

R2, Comment 10 — Mood and online response styles

“Data for all variables were collected online; does this reflect differences in student mood and response style?”

Response: We agree that online self-report cannot separate substantive covariance from momentary mood or response style. The revised Limitations section now discusses mood, acquiescence, inattentive responding, and common method bias. We recommend temporal separation, marker-variable or common-latent-factor approaches, app logs, and accelerometer measures in future work.

R2, Comment 11 — Flow diagram and subgroup comparisons

“A flow diagram is required to illustrate research activities and measurements. Are there differences between students based on gender, academic year, or duration of app usage (three months versus more than one year)?”

Response: Thank you. We added a flow diagram showing recruitment, completed submissions, exclusions, final measurements, and analyses. We also conducted the requested comparisons. No significant differences were found across gender, academic year, or the four app-use-duration categories for GAMEX, EI, EHF, IU, or EA (all p > .15; small effect sizes). The direct comparison between less than three months and more than one year was also non-significant for GAMEX (p = .330), EI (p = .895), EHF (p = .506), IU (p = .174), and EA (p = .553). See Fig 1 and Demographic and app-use group comparisons.

R2, Comment 12 — Improve Fig 1

“Improve Fig. 1 by enlarging the text. Use either the full term or the abbreviation consistently in the figure caption.”

Response: We agree. The conceptual model is now displayed at a larger manuscript width, and the caption defines all abbreviations consistently. Because the new participant-flow diagram is placed first, the conceptual model is renumbered automatically in the compiled revision.

R2, Comment 13 — Comparison with published studies

“Compare the results with findings from relevant published studies.”

Response: Thank you; this prompted a substantial revision of the Discussion. We now compare the GAMEX–adherence result with randomized trials and meta-analyses showing both positive and limited-maintenance effects. The identity section compares supportive longitudinal and intervention findings with a study in which changes in identity and accelerometer-measured exercise did not covary reliably. The IU section now draws on experimental information-seeking evidence and discusses competing explanations. Ten relevant references were added across the manuscript. See all four substantive Discussion subsections.

R2, Comment 14 — Primary findings in the Conclusion

“Address the data analysis of primary findings in the conclusion section.”

Response: We agree. The Conclusion now reports the GAMEX–EA association, all three indirect-effect estimates with 95% confidence intervals, and the GAMEX × IU coefficient, followed immediately by the cross-sectional interpretation limit.

R2, Comment 15 — Future research

“Suggestions for future research should be addressed at the end of the conclusion section.”

Response: Thank you. The final sentences now call for multi-wave tests of alternative causal orderings, experiments separating predictable feedback from uncertain rewards, objective app-log and accelerometer outcomes, and tests of IU-informed personalization before any design recommendations are adopted.

Closing

We are grateful to the Academic Editor and both reviewers for comments that led us to reconsider the strength of our claims, make the data and procedures easier to audit, and place the results more carefully within the existing literature. We hope that the revised manuscript now addresses the concerns fully and meets PLOS ONE’s publication criteria.

Sincerely,

Jingjing Song, Jiaying Li, and Dan Li

# Peer review: Decision Letter 1

**DOI:** 10.1371/journal.pone.0353100.r003

**Authors:** Amir Karimi

8 Sep 2026

Dear Dr. Song,

Thank you for submitting your manuscript to PLOS One. After careful consideration, we feel that it has merit but does not fully meet PLOS One’s publication criteria as it currently stands. Therefore, we invite you to submit a revised version of the manuscript that addresses the points raised during the review process.

Please submit your revised manuscript by  Oct 08 2026 11:59PM. If you will need more time than this to complete your revisions, please reply to this message or contact the journal office at plosone@plos.org. When you're ready to submit your revision, log on to https://www.editorialmanager.com/pone/ and select the 'Submissions Needing Revision' folder to locate your manuscript file.

- A letter that responds to each point raised by the academic editor and reviewer(s). You should upload this letter as a separate file labeled 'Response to Reviewers'.
- A marked-up copy of your manuscript that highlights changes made to the original version. You should upload this as a separate file labeled 'Revised Manuscript with Track Changes'.
- An unmarked version of your revised paper without tracked changes. You should upload this as a separate file labeled 'Manuscript'.

If you would like to make changes to your financial disclosure, please include your updated statement in your cover letter. Guidelines for resubmitting your figure files are available below the reviewer comments at the end of this letter.

If applicable, we recommend that you deposit your laboratory protocols in protocols.io to enhance the reproducibility of your results. Protocols.io assigns your protocol its own identifier (DOI) so that it can be cited independently in the future. For instructions see: https://journals.plos.org/plosone/s/submission-guidelines#loc-laboratory-protocols. Additionally, PLOS One offers an option for publishing peer-reviewed Lab Protocol articles, which describe protocols hosted on protocols.io. Read more information on sharing protocols at https://plos.org/protocols?utm_medium=editorial-email&utm_source=authorletters&utm_campaign=protocols.

As the corresponding author, your ORCID iD is verified in the submission system and will appear in the published article. PLOS supports the use of ORCID, and we encourage all coauthors to register for an ORCID iD and use it as well. Please encourage your coauthors to verify their ORCID iD within the submission system before final acceptance, as unverified ORCID iDs will not appear in the published article. *Only*  the individual author can complete the verification step; PLOS staff *cannot*  verify ORCID iDs on behalf of authors.

We look forward to receiving your revised manuscript.

Kind regards,

Amir Karimi, PhD

Academic Editor

PLOS One

Journal Requirements:

If the reviewer comments include a recommendation to cite specific previously published works, please review and evaluate these publications to determine whether they are relevant and should be cited. There is no requirement to cite these works unless the editor has indicated otherwise.

Please review your reference list to ensure that it is complete and correct. If you have cited papers that have been retracted, please include the rationale for doing so in the manuscript text, or remove these references and replace them with relevant current references. Any changes to the reference list should be mentioned in the rebuttal letter that accompanies your revised manuscript. If you need to cite a retracted article, indicate the article’s retracted status in the References list and also include a citation and full reference for the retraction notice.

[Note: HTML markup is below. Please do not edit.]

Reviewers' comments:

Reviewer's Responses to Questions

**Comments to the Author**

Reviewer #2: (No Response)

**********

2. Is the manuscript technically sound, and do the data support the conclusions?

Reviewer #2: Partly

**********

3. Has the statistical analysis been performed appropriately and rigorously?

Reviewer #2: Yes

**********

4. Have the authors made all data underlying the findings in their manuscript fully available?

The PLOS Data policy

Reviewer #2: (No Response)

**********

5. Is the manuscript presented in an intelligible fashion and written in standard English?

Reviewer #2: (No Response)

**********

Reviewer #2: Manuscript Number: PONE-D-26-28180R1

Despite the authors’ efforts to incorporate the reviewers’ comments, the manuscript has improved; however, minor issues remain that should be addressed before further consideration by PLOS One.

1. Excessive dashes (-) appear in the background of the abstract and should be removed.

2. The introduction should be expanded and divided into three paragraphs.

3. Gender details are still missing in Fig. 1 and/or Table 1.

4. There are two captions for Fig. 1; this should be resolved.

5. The word “abbreviations” should be used in figure captions where full names have been provided.

6. Increase the size and clarity of Figures 2, 3, and 4, including all internal and external text.

7. Discuss results in the context of relevant published studies. It should be specified whether findings are

consistent with or contradict the existing literature.

8. The conclusion section should begin with a brief statement of the study’s objective and conclude with its potential applications.

**********

what does this mean?). If published, this will include your full peer review and any attached files.

If you choose “no”, your identity will remain anonymous but your review may still be made public.

**Do you want your identity to be public for this peer review?**  For information about this choice, including consent withdrawal, please see our Privacy Policy

Reviewer #2: No

**********

[NOTE: If reviewer comments were submitted as an attachment file, they will be attached to this email and accessible via the submission site. Please log into your account, locate the manuscript record, and check for the action link "View Attachments". If this link does not appear, there are no attachment files.]

To ensure your figures meet our technical requirements, please review our figure guidelines: https://journals.plos.org/plosone/s/figures

You may also use PLOS’s free figure tool, NAAS, to help you prepare publication quality figures: https://journals.plos.org/plosone/s/figures#loc-tools-for-figure-preparation.

NAAS will assess whether your figures meet our technical requirements by comparing each figure against our figure specifications.

# Author comment: Author response to Decision Letter 2

**DOI:** 10.1371/journal.pone.0353100.r004

9 Sep 2026

Response to Reviewer

Manuscript PONE-D-26-28180R1

Gameful experience, exercise identity, and exercise adherence among Chinese university students: a moderated chain mediation model

Dear Dr. Karimi and Reviewer #2,

Thank you for the careful second-round review and for recognizing the improvement made in the previous revision. We appreciate the opportunity to address the remaining presentation and reporting issues. We have revised each point and describe the changes below. Page and figure numbering refer to the clean revised manuscript.

Response to Reviewer #2

Comment 1 — Dashes in the Abstract background

Reviewer comment: The background section of the Abstract contains too many dashes; please remove them.

Response: Thank you for identifying this stylistic issue. We removed the parenthetical dash construction from the Abstract background. The definition of gameful experience is now incorporated with a conventional comma construction, which improves readability without changing the meaning. See the Abstract, Background.

Comment 2 — Expansion and organization of the Introduction

Reviewer comment: The Introduction should be expanded and divided into three independent paragraphs.

Response: We agree. The Introduction has been expanded and reorganized into three substantive paragraphs before the hypotheses. Paragraph 1 establishes the physical-activity problem, reviews evidence on gamified fitness applications, and identifies the gap concerning sustained exercise and subjective gameful experience. Paragraph 2 develops the exercise-identity and habit-formation rationale and explains their proposed sequence. Paragraph 3 develops IU as a boundary condition, presents a competing explanation, describes the Chinese university context, and states the study model. The revised structure provides a clearer progression from background to the specific research question.

Comment 3 — Gender information in Fig 1 or Table 1

Reviewer comment: Gender-related sample information is still missing from Fig 1 or Table 1.

Response: Thank you. Although gender counts were reported in the Participants subsection, we agree that they should also be visible in the requested display. The analytic-sample box in Fig 1 now reports 148 men (49.3%) and 152 women (50.7%), and the same information is repeated once in the Fig 1 caption.

Comment 4 — Duplicate title or caption in Fig 1

Reviewer comment: Fig 1 contains two sets of titles/captions and this should be corrected.

Response: We agree. Fig 1 now has one journal-formatted caption only. Descriptive information about the participant flow and gender distribution has been consolidated into that single caption, and no separate or duplicate figure title is retained.

Comment 5 — Abbreviations in figure captions

Reviewer comment: If abbreviations appear in a figure caption, please use “abbreviations” to explain their full forms.

Response: Thank you. Every caption containing shortened terms now includes a clearly labeled “Abbreviations:” statement. The captions define GAMEX, EI, EHF, IU, EA, and, where relevant, SD. The format has been applied consistently to Figs 1–4.

Comment 6 — Size and clarity of Figs 2–4

Reviewer comment: Please enlarge Figs 2, 3, and 4, including all text inside and outside the figures.

Response: We agree. Figs 2–4 are now displayed at 98% of the manuscript text width. For Figs 2 and 4, unused white margins were removed so that the model boxes, path labels, and coefficients occupy substantially more of the available width. Their TIFF files are RGB, LZW-compressed, 600 dpi, and 3274 × 1222 pixels. Fig 3 is supplied as an RGB TIFF at 300 dpi and 1884 × 1282 pixels and is also enlarged in the manuscript. We additionally corrected the EHF–EA significance marker in Fig 4 to 0.426*** so that it matches Table 5 (p < .001).

Comment 7 — Explicit comparison with published studies

Reviewer comment: Discuss the results in relation to published research and state clearly whether the findings agree or conflict with existing evidence.

Response: Thank you. We made the comparisons more explicit in the Discussion. The GAMEX–adherence result is now described as agreeing with studies that link enjoyment, competence, and gamification-feature richness with continued use or adherence intention. We then contrast it with trials reporting inconsistent maintenance effects and discuss differences in follow-up duration, app features, outcomes, and assigned gamification versus subjective gameful experience. The exercise-identity section now identifies both agreement with longitudinal and intervention evidence and disagreement with evidence from behavioral weight-loss treatment, followed by possible contextual and measurement explanations. These revisions appear in “Gameful experience and exercise adherence” and “The mediating role of exercise identity.”

Comment 8 — Aim and practical implications in the Conclusion

Reviewer comment: The Conclusion should begin with a brief statement of the study aim and end with potential practical applications.

Response: We agree. The Conclusion now opens by stating that the study examined indirect associations of gameful experience with adherence through exercise identity and habit formation and tested IU as a qualifier of the GAMEX–EI association. It ends with a cautious practical implication: if the pattern is replicated longitudinally or experimentally, researchers and developers could test progress-visibility, identity-supportive, and stable-context features. We retain the important qualification that IU-sensitive personalization is an experimental option rather than an established implementation recommendation.

Closing

We thank the reviewer again for these precise suggestions. They improved the organization of the Introduction, the transparency of the sample description, the readability and consistency of the figures, and the balance of the Discussion and Conclusion. We hope that the revised manuscript now addresses all remaining concerns.

Sincerely,

Jingjing Song, Jiaying Li, and Dan Li

Attachment Submitted filename: PONE-D-26-28180R1_Response_to_Reviewers.pdf

# Editor report: Decision Letter 2

**DOI:** 10.1371/journal.pone.0353100.r005

**Authors:** Amir Karimi

15 Sep 2026

<p>Gameful experience, exercise identity, and exercise adherence among Chinese university students: a moderated chain mediation model

PONE-D-26-28180R2

Dear Dr. Song,

We’re pleased to inform you that your manuscript has been judged scientifically suitable for publication and will be formally accepted for publication once it meets all outstanding technical requirements.

Within one week, you’ll receive an e-mail detailing the required amendments. When these have been addressed, you’ll receive a formal acceptance letter and your manuscript will be scheduled for publication.

An invoice will be generated when your article is formally accepted. Please note, if your institution has a publishing partnership with PLOS and your article meets the relevant criteria, all or part of your publication costs will be covered. Please make sure your user information is up-to-date by logging into Editorial Manager at Editorial Manager® and clicking the ‘Update My Information' link at the top of the page. For questions related to billing, please contact billing support.

If your institution or institutions have a press office, please notify them about your upcoming paper to help maximize its impact. If they’ll be preparing press materials, please inform our press team as soon as possible -- no later than 48 hours after receiving the formal acceptance. Your manuscript will remain under strict press embargo until 2 pm Eastern Time on the date of publication. For more information, please contact onepress@plos.org.

Kind regards,

Amir Karimi, PhD

Academic Editor

PLOS One

Additional Editor Comments (optional):

Reviewers' comments:

# Editor report: Acceptance letter

**DOI:** 10.1371/journal.pone.0353100.r006

**Authors:** Amir Karimi

PONE-D-26-28180R2

PLOS One

Dear Dr. Song,

I'm pleased to inform you that your manuscript has been deemed suitable for publication in PLOS One. Congratulations! Your manuscript is now being handed over to our production team.

At this stage, our production department will prepare your paper for publication. This includes ensuring the following:

* All references, tables, and figures are properly cited

* All relevant supporting information is included in the manuscript submission,

* There are no issues that prevent the paper from being properly typeset

You will receive further instructions from the production team, including instructions on how to review your proof when it is ready. Please keep in mind that we are working through a large volume of accepted articles, so please give us a few days to review your paper and let you know the next and final steps.

Lastly, if your institution or institutions have a press office, please let them know about your upcoming paper now to help maximize its impact. If they'll be preparing press materials, please inform our press team within the next 48 hours. Your manuscript will remain under strict press embargo until 2 pm Eastern Time on the date of publication. For more information, please contact onepress@plos.org.

You will receive an invoice from PLOS for your publication fee after your manuscript has reached the completed accept phase. If you receive an email requesting payment before acceptance or for any other service, this may be a phishing scheme. Learn how to identify phishing emails and protect your accounts at https://explore.plos.org/phishing.

If we can help with anything else, please email us at customercare@plos.org.

Thank you for submitting your work to PLOS One and supporting open access.

Kind regards,

PLOS One Editorial Office Staff

on behalf of

Dr. Amir Karimi

Academic Editor

PLOS One
